# Early Use of Molecular Diagnostics in the Emergency Department Reduces All-Cause Mortality in Patients with Pneumonia at Risk of Multidrug-Resistant Pathogens

**DOI:** 10.3390/diagnostics16193243

**Published:** 2026-10-08

**Authors:** Lorenzo Pelagatti, Francesca Mangani, Antonio Susanna, Lorenzo Corbetta, Michele Spinicci, Alessandro Bartoloni, Peiman Nazerian, Gian Maria Rossolini, Simone Vanni

**Affiliations:** 1 Department of Experimental and Clinical Medicine, University of Florence, Largo G.A. Brambilla, 3, 50134 Florence, Italy; francesca.mangani@unifi.it (F.M.); antonio.susanna@unifi.it (A.S.); lorenzo.corbetta@unifi.it (L.C.); michele.spinicci@unifi.it (M.S.); alessandro.bartoloni@unifi.it (A.B.); gianmaria.rossolini@unifi.it (G.M.R.); simone.vanni@unifi.it (S.V.); 2 Emergency Department, Careggi University Hospital, 50134 Florence, Italy; nazerianp@aou-careggi.toscana.it; 3 Microbiology and Virology Unit, Careggi University Hospital, 50134 Florence, Italy; 4 Interventional Pulmonology Unit, Careggi University Hospital, 50134 Florence, Italy; 5 Infectious and Tropical Diseases Unit, Careggi University Hospital, 50134 Florence, Italy; 6 High Dependency Unit, Careggi University Hospital, 50134 Florence, Italy

**Keywords:** multiplex PCR, molecular syndromic panels, pneumonia, emergency department, mortality, antimicrobial stewardship

## Abstract

**Background/Objectives**: Patients with community-acquired pneumonia at risk of multidrug-resistant pathogens (CAP-MDR) represent a high-risk population in which empiric therapy is frequently inadequate. Evidence on the impact of multiplex syndromic panels (MSP) on clinical outcomes in CAP-MDR pneumonia remains limited, particularly in the emergency department (ED). **Methods**: We conducted a single-center observational pre–post study including patients presenting to the ED with CAP-MDR. Patients were managed either with early MSP-guided therapy (prospective cohort) or standard empiric treatment (retrospective cohort). The primary outcome was 30-day mortality. Analyses were performed in the overall population and subsequently in a propensity score–matched cohort. **Results**: A total of 718 patients were included (MSP n = 128; no-MSP n = 590). Thirty-day mortality was significantly lower in the MSP group (22.7% vs. 31.9%; *p* = 0.04). At multivariable logistic regression, MSP use was independently associated with reduced mortality (OR 0.51, 95% CI 0.30–0.88; *p* = 0.016), confirmed by Cox analysis (HR 0.60, 95% CI 0.36–0.99; *p* = 0.04). Kaplan–Meier analysis showed improved survival (log-rank *p* = 0.002). After propensity score-matching (MSP n = 128; no-MSP n = 128), mortality remained lower in the MSP group (22.7% vs. 35.1%; *p* = 0.02). MSP use was independently associated with reduced mortality (OR 0.45, 95% CI 0.20–0.99; *p* = 0.04), confirmed by Cox regression (HR 0.50, 95% CI 0.31–0.82; *p* = 0.006). **Conclusions**: Early use of MSP in patients with CAP-MDR was associated with a significant reduction in 30-day mortality. Rapid molecular diagnostics may provide the greatest clinical benefit when applied early in high-risk populations in which empiric therapy is frequently inadequate.

## 1. Introduction

Pneumonia is a leading cause of hospital admission and of infection-related mortality worldwide [1]. Within the spectrum of community-acquired pneumonia (CAP), infections caused by pathogens typically associated with healthcare settings are increasingly recognized (CAP-MDR) [2]. These organisms often exhibit resistance to one or more antibiotic classes, rendering standard empiric regimens for community-acquired pneumonia inadequate [1,2,3].

Delayed initiation of appropriate antibiotic therapy is associated with increased mortality and overall worse outcomes [3]. Therefore, empiric treatment is usually started to cover the broadest spectrum of infecting pathogens in relation to local epidemiology and possibly downgraded upon receipt of microbiological results [2]. This approach has contributed to increased use of broad-spectrum antibiotics, with consequent selection of antimicrobial resistance. Moreover, conventional diagnostic methods based on culture may fail to identify a causative pathogen in 25–60% of cases [4].

Molecular syndromic panels (MSPs) enable rapid identification of respiratory pathogens within a few hours and generally provide higher sensitivity than conventional microbiology [5,6,7]. Their use is increasingly common in HAP (hospital-acquired pneumonia) and VAP (ventilator-acquired pneumonia), particularly in settings with high antimicrobial resistance, and has recently been explored also in CAP-MDR patients presenting to the emergency department (ED) [7,8]. However, although MSPs have consistently demonstrated benefits for antimicrobial stewardship, evidence of a meaningful impact on clinical outcomes remains limited [9,10,11,12,13,14,15,16,17,18,19,20], and their use is not yet standardized in international guidelines [21].

Most available studies have been conducted in intensive care settings, often with heterogeneous populations [9,22,23,24,25]. Furthermore, in conditions such as CAP, empiric therapy often provides adequate pathogen coverage, potentially attenuating the observable impact of molecular diagnostics on clinical outcomes [26,27,28].

In principle, the use of MSP in patients with CAP-MDR could offer greater clinical benefit by enabling earlier identification of pathogens resistant to the standard empiric therapy for CAP, thereby facilitating timely initiation of appropriate therapy in these cases [7,29]. Accordingly, this study evaluated whether an early use (within 24 h from ED triage) of an MSP could improve meaningful patient-centric clinical outcomes in patients presenting to the ED with CAP-MDR as the primary reason for hospital admission.

## 2. Materials and Methods

The HCAPPED II study was approved by the competent Ethical Committee (Comitato Etico Area Vasta Centro, CEAVC 26198_oss) and registered on ClinicalTrials.gov (NCT06506617, 16 July 2024).

This was a single-center, non-profit, observational pre–post study, including a prospective cohort (1 June 2024–31 December 2025) and a retrospective control cohort (1 January 2023–31 May 2024). The study population consisted of patients with diagnosis of CAP-MDR at ED admission. Both cohorts underwent conventional microbiological diagnostics (nasopharyngeal swab, urinary antigens, blood cultures). In the prospective cohort, lower respiratory samples (endotracheal aspirate, ETA; bronchial aspirate, BAS; bronchoalveolar lavage, BAL) were collected within 24 h of initial ED presentation and then analysed using an MSP (BIOFIRE^®^ FILMARRAY^®^ Pneumonia Plus Panel, bioMérieux, 376, Chemin de l’Orme, Marcy-l’Étoile, France) (MSP cohort). Empiric antibiotic therapy was initiated immediately according to institutional recommendations and subsequently reassessed as soon as MSP results became available, allowing early escalation, de-escalation, or targeted modification when indicated. In contrast, patients in the retrospective cohort were managed according to the same empiric treatment recommendations, with subsequent therapeutic adjustments based exclusively on conventional microbiological results and clinical evolution. (Supplementary Material—Figure S1) [2,26,27,28].

### 2.1. Objective

The primary objective was to evaluate the impact of early use of molecular diagnostics on the clinical outcome in the prospective cohort in terms of 30-day all-cause mortality vs. the retrospective cohort. In the retrospective cohort, the 30-day all-cause mortality was ascertained from electronic medical records and, when necessary, by telephone follow-up.

Secondary objectives included comparisons between the two cohorts in terms of: (a) mean length of hospital stay among survivors; (b) 30-day readmission rate; (c) rate of identification of pathogens putatively responsible for CAP-MDR within the first 7 days from ED presentation (triage) using all available diagnostic methods (MSP, culture, antigen detection, and serology); and (d) rate of escalation of supportive care within 30 days from ED presentation, defined as the initiation of invasive or non-invasive mechanical ventilation and/or vasoactive support in patients who did not require these interventions at baseline. Additionally, in the MSP cohort, we evaluated the rate of modification of antibiotic therapy (either escalation or de-escalation) compared with local empiric treatment recommendations within 24 h of ED presentation. Escalation was defined as broadening the antibiotic spectrum, either by adding another agent or by switching to a broader spectrum drug presumably active against the identified pathogen. De-escalation was defined as narrowing the antibiotic spectrum by discontinuing unnecessary agents or switching to a narrower spectrum drug.

Safety outcomes included complications related to the collection of lower respiratory samples (ETA, BAS or BAL), and the time in hours from collection of respiratory samples to administration of pathogen-targeted antibiotic therapy; this variable was analyzed as a continuous measure, with time intervals calculated based on timestamps recorded in the electronic medical records.

### 2.2. Study Population and Setting

The study was conducted at the Department of Emergency Medicine and High Dependency Unit (HDU) of the Florence Careggi University Hospital, a large hospital serving a local population of approximately 300,000 inhabitants and acting as a referral center for nearly 1,600,000 individuals, in collaboration with the Microbiology and Virology and the Infectious and Tropical Diseases Units of the same hospital (see Supplementary Materials).

### 2.3. Enrollment Criteria

#### 2.3.1. Inclusion Criteria

In the prospective cohort (MSP cohort), all patients aged ≥18 years presenting to the ED with a clinical and radiological diagnosis of CAP-MDR, with at least one of the severity criteria at presentation as defined in the HCAPPED-I study population [7], and undergoing respiratory sampling (BAL, BAS, ETA) processed with an MSP within the first 24 h of ED admission were considered eligible.

Severity criteria included: (i) requirement for high-flow oxygen therapy (e.g., VM35, FiO_2_ > 0.35, reservoir mask, high-flow nasal cannula-HFNC-or non-invasive ventilation-NIV); (ii) endotracheal intubation (IOT); (iii) SOFA score ≥ 2 (respiratory component) or Pneumonia Severity Index (PSI) > 85 (e.g., age > 75 + respiratory impairment); (iv) Horowitz index (PaO_2_/FiO_2_) < 200.

Pneumonia was defined according to IDSA guidelines as the presence of a new pulmonary infiltrate on chest radiograph associated with at least two of the following: fever > 38 °C, leukocytosis or leukopenia, and purulent respiratory secretions [1]. CAP-MDR was defined by the presence of at least one of the following risk factors: (a) hospitalization in an acute-care hospital for ≥2 days within the 90 days preceding symptom onset; (b) residence in a nursing home or long-term care facility; (c) recent (within 30 days) intravenous antibiotic therapy, chemotherapy, or wound care; (d) attendance at a hospital or haemodialysis clinic within the previous 30 days [30].

Patients in the retrospective cohort were required to meet the same inclusion criteria, except for the requirement of undergoing respiratory sampling and meeting the severity criteria.

#### 2.3.2. Exclusion Criteria

Patients were excluded if they did not provide informed consent to participate in the study. Additional exclusion criteria included age younger than 18 years or older than 90 years, pregnancy, and an estimated life expectancy of less than three months. Patients with severe baseline disability, defined as a modified Rankin Scale (mRS) score ≥ 5, were also excluded [31]. Finally, patients presenting with CAP without risk factors for MDR pathogens were not eligible for inclusion in the study.

### 2.4. Microbiological Investigations

See Supplementary Materials and Table S1 (Section 2).

### 2.5. Sample Size

Assuming a two-sided alpha of 5%, 80% power, a 30-day mortality of at least 20% and an expected hazard ratio of 0.65 between the prospective and retrospective cohort [32], approximately 210 patients would be required overall, corresponding to 105 patients per group, according to Schoenfeld’s method [33]. Considering a 10% drop-out or missing data, the planned sample size was defined as at least 230 patients overall, 115 per cohort.

### 2.6. Statistical Analysis

The impact of early use of molecular diagnostics on patient prognosis was assessed by comparing 30-day all-cause mortality between patients receiving MSP-guided antibiotic therapy (prospective cohort) and those treated with standard empirical therapy (retrospective cohort). The outcome was analyzed both as a dichotomous variable and as event proportions across groups. Between-group differences were evaluated using the chi-square or Fisher’s exact test, as appropriate. The association between early MSP use and 30-day mortality was further assessed using multivariable logistic regression, with estimation of odds ratios (ORs) and 95% confidence intervals (CIs), adjusting for clinically relevant confounders.

Time-to-event analysis was performed using Kaplan–Meier curves and the log-rank test, while a Cox proportional hazards model was used to estimate hazard ratios (HRs) with 95% CIs, including relevant clinical covariates.

Patient data were recorded in a dedicated case report form (CRF). Each patient was pseudo-anonymized using a numerical identification code. The principal investigator was responsible for overseeing data collection and data integrity.

The normality of the distribution of continuous variables was assessed using Q–Q plots and the Shapiro–Wilk test. Quantitative variables were reported as mean and standard deviation (SD). Qualitative variables were described using absolute and relative frequencies.

Comparisons between the two study groups were performed using Student’s *t*-test for normally distributed variables or the Mann–Whitney U test for non-normally distributed variables. For categorical variables, Fisher’s exact test or the chi-square test was used as appropriate. A two-sided *p*-value <0.05 was considered statistically significant.

Given the observational pre–post design and the potential baseline differences between the retrospective control group and the prospective MSP cohort, a propensity score matching (PSM) analysis was performed to reduce confounding. Variables included in the propensity score model were selected a priori based on clinical relevance and previously identified confounders in the literature rather than on statistical significance alone [34]. Patients were matched 1:1 using nearest-neighbor matching based on age, sex, Horowitz index, active cancer, immunosuppression, chronic heart failure, and recent hospitalization (≥2 days within the previous 90 days) [35,36,37,38,39]. After matching, the same analyses performed in the overall cohort were repeated in the matched population.

All statistical analyses were performed using IBM SPSS Statistics software (version 29.0, IBM Corp., Armonk, NY, USA).

## 3. Results

**Figure 1:**
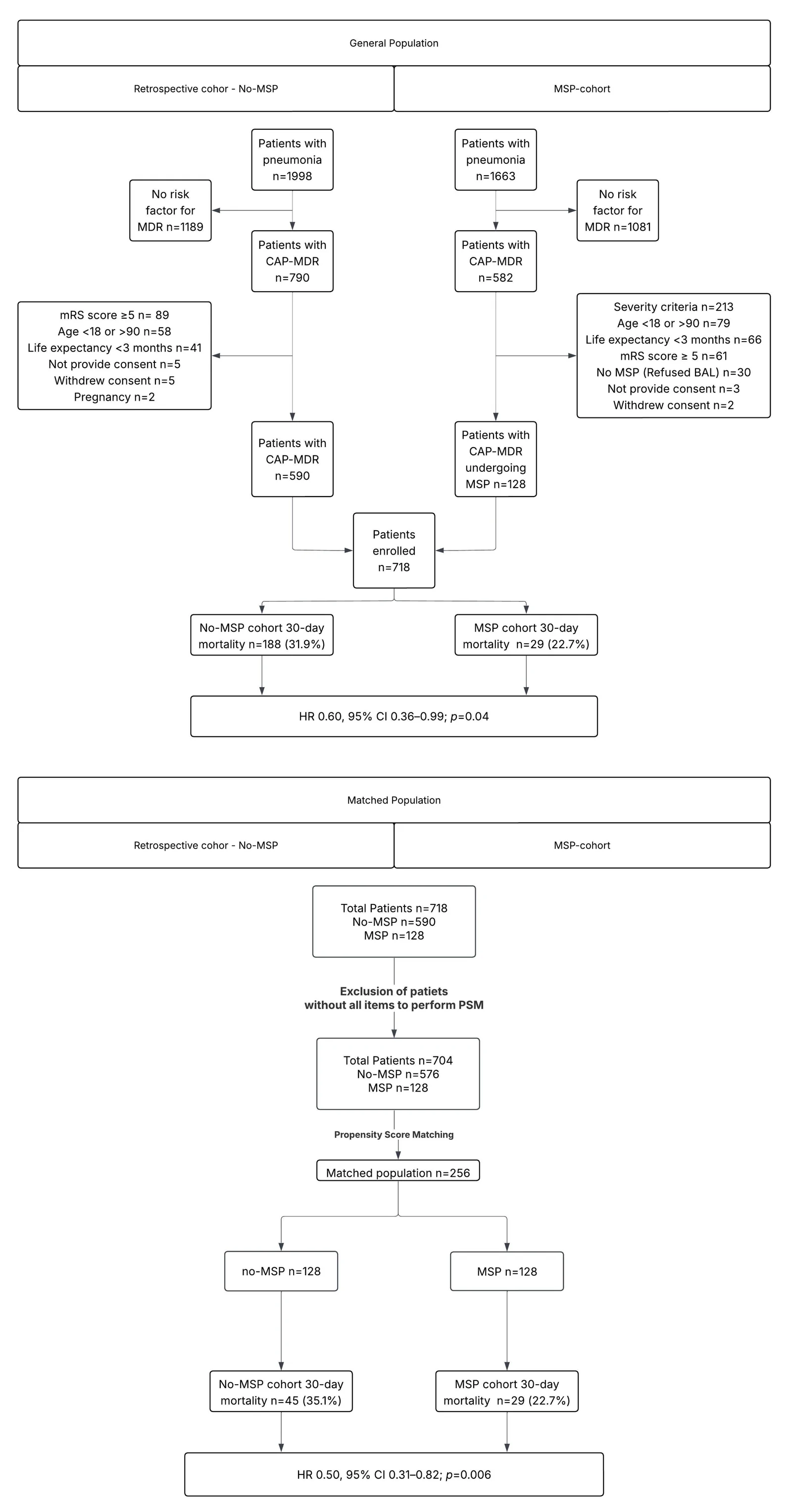
Study flow diagram and propensity score matching. MDR: multidrug-resistant; CAP-MDR: community-acquired pneumonia at risk for multidrug-resistant pathogens; MSP: molecular syndromic panel; PSM: propensity score matching; OR: odds ratio; CI: confidence interval; HR: hazard ratio; mRS: modified Rankin Scale.

A total of 718 CAP-MDR patients presenting to the ED of our hospital were enrolled during the study period, including a prospective cohort of patients subjected to fast microbiological analysis by an MSP (MSP cohort), in addition to the conventional diagnostic workflow (1 June 2024–31 December 2025; n = 128) and a retrospective cohort of patients (no-MSP cohort) subjected only to the conventional diagnostic workflow (1 January 2023–31 May 2024; n = 590) (Figure 1).

The mean age of the study population was 75.7 ± 13.8 years, and 38.0% were female (273/718). Patients in the MSP cohort were significantly younger compared with those in the no-MSP cohort (72.1 ± 15.3 vs. 76.5 ± 13.3 years; *p* = 0.02) and exhibited less frequently some comorbidities, including congestive heart failure and atrial fibrillation (*p* < 0.01 and *p* = 0.04, respectively) (Table 1). They also reported more frequent recent hospitalization (≥2 days within 90 days), nursing home residency, and recent antibiotic therapy compared with those in the retrospective/non-MSP cohort (*p* = 0.01, *p* = 0.03, and *p* = 0.03, respectively) (Table 2). Concerning vital parameters at ED admission, patients in the MSP cohort presented with significantly higher respiratory rate and FiO_2_ requirements compared with those in the no-MSP cohort (*p* < 0.01 for both) (Table 3). Regarding laboratory and arterial blood gas parameters, significant differences between the two cohorts were observed for pH, pO_2_, pCO_2_, and bicarbonate levels (all *p* < 0.01) (Table S2—Supplementary Materials).

### 3.1. Primary Outcome

The overall 30-day mortality rate was 30.2% (217/718): 155 (21.6%) died during hospitalization and 62 (8.6%) after discharge, within 30 days of ED admission.

**Figure 2:**
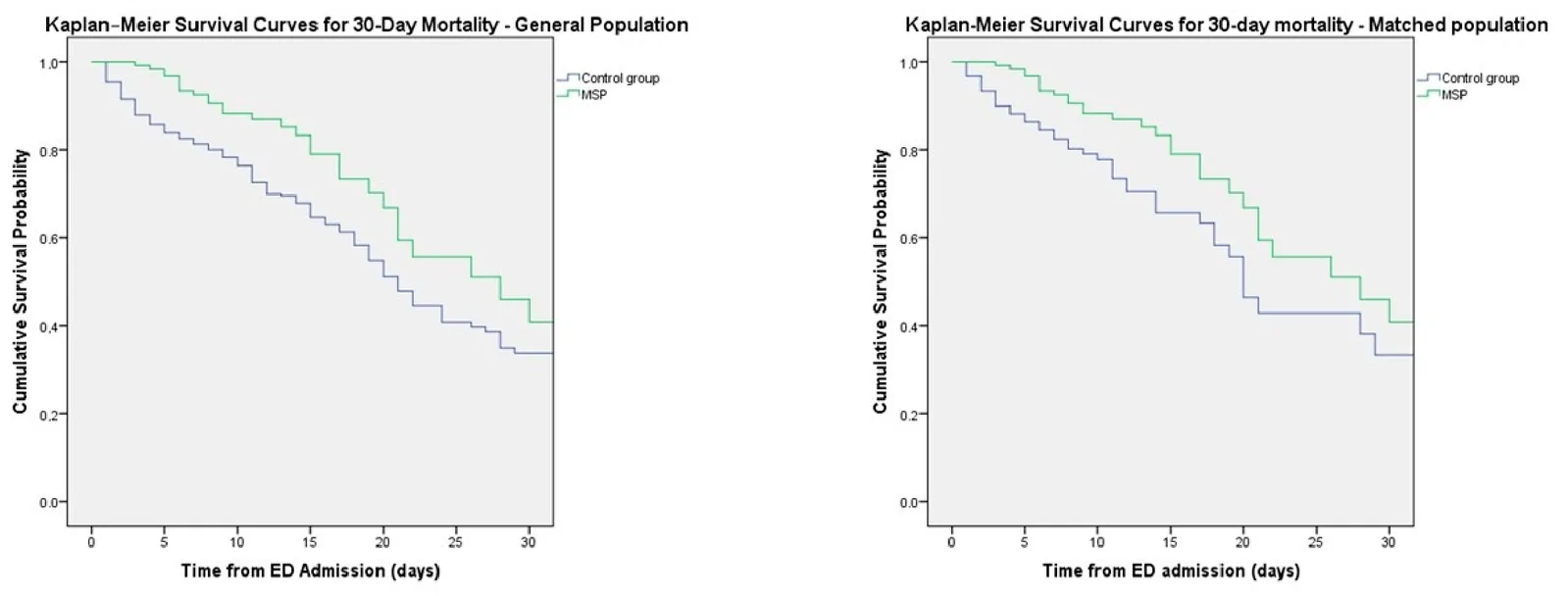
(**Left panel**) Kaplan–Meier survival curves for 30-day mortality in the overall population, showing significantly improved survival in the MSP group compared with controls (log-rank Mantel–Cox *p* = 0.002). (**Right panel**) Kaplan–Meier survival curves for 30-day mortality in the matched population. Survival was significantly higher in the MSP cohort compared with the control group (log-rank Mantel–Cox *p* = 0.008).

Thirty-day mortality was significantly lower in the MSP cohort compared with the no-MSP cohort (22.7% [29/128] vs. 31.9% [188/590]; *p* = 0.04). Kaplan–Meier analysis demonstrated significantly improved survival in the MSP cohort compared with the no-MSP cohort (log-rank Mantel–Cox *p* = 0.002) (Figure 2—left panel). In the multivariable logistic regression analysis, early use of MSP was independently associated with a reduced risk of mortality (OR 0.51, 95% CI 0.30–0.88; *p* = 0.016). Among covariates, increasing age (OR 1.07 per year, 95% CI 1.04–1.09; *p* < 0.001), higher respiratory rate (OR 1.06, 95% CI 1.02–1.10; *p* = 0.001), and lower systolic blood pressure (OR 0.98, 95% CI 0.97–0.99; *p* < 0.001) were independently associated with mortality. A Cox proportional hazards model confirmed these findings, showing that MSP use was independently associated with a reduced risk of death (HR 0.60, 95% CI 0.36–0.99; *p* = 0.04) (Table S3—Supplementary Materials).

In a sensitivity analysis restricted to retrospective controls fulfilling the clinical severity criteria used for enrollment in the prospective cohort, 541 of 590 control patients were eligible. Thirty-day mortality remained significantly lower in the MSP group (29/128, 22.7%) than in the restricted control cohort (184/541, 34.0%), corresponding to an OR of 0.57 (95% CI, 0.36–0.89; *p* = 0.014).

### 3.2. Secondary Outcomes

Among secondary outcomes, significant differences between the MSP and the no-MSP cohort were observed in LOS (12.9 ± 10.0 vs. 9.9 ± 9.1 days; *p* = 0.01) and thirty-day readmission rates (9.6% vs. 19.4%; *p* = 0.018). Following multivariable logistic regression, early MSP use was independently associated with a reduced risk of 30-day readmission (OR 0.38, 95% CI 0.18–0.83; *p* = 0.014), while no other variables (among comorbidities and vital signs) were significantly associated with that outcome.

Identification of the putative CAP-MDR etiology within the first 7 days from inclusion, considering all diagnostic methods, was significantly higher in the MSP compared with the no-MSP cohort (94.8% vs. 41.3%; *p* < 0.001). The results of conventional microbiological tests were similar in the two groups (Table S4—Supplementary Materials).

A trend was observed in the need for escalation of ventilatory support between the MSP and no-MSP groups (3.9% vs. 8.6%; *p* = 0.09). No significant differences were observed in the need for initiation or escalation of vasoactive (vasopressor) support between the MSP and no-MSP groups (3.9% vs. 3.6%, *p* = 0.79).

MSP results and safety outcomes are reported in the Supplementary Material.

### 3.3. MSP and Cultures Results

In the MSP cohort, respiratory samples were predominantly BAL specimens (119/128, 93.0%), while BAS and ETA accounted for 4/128 (3.1%) and 5/128 (3.9%) of samples, respectively. MSP testing identified pathogens in 90.6% of cases. Polymicrobial infections were detected in 62.5% of patients, while resistance genes were identified in 35.2%. The mean time from ED admission to PCR testing was 9.1 ± 6.7 h. MSP results supported treatment modification in 85/128 patients (66.4%), including escalation in 51/128 (39.9%) and de-escalation in 34/128 (26.5%), whereas no modification would have been indicated in 43/128 cases (33.6%). Pathogens and resistance genes have been reported in Table S5—Supplementary Materials.

Respiratory cultures were positive in 85/128 cases (66.4%). Among these, 46/128 (35.9%) were caused by multidrug-resistant (MDR) pathogens, similar to MSP testing, while the remaining isolates were susceptible to standard antibiotics.

At the microorganism level, MSP yielded 156 bacterial detections. Of these, 68 (43.6%) were also identified by conventional respiratory culture, whereas 88 (56.4%) were not recovered by culture.

Carbapenemase genes were detected in 18 patients. Respiratory cultures were positive in 17/18 (94.4%), with 100% agreement between carbapenemase gene detection and the corresponding phenotypic carbapenem-resistant profile among culture-positive cases.

No major complications related to respiratory sample acquisition were observed. Only minor adverse events were reported, such as cough and transient hypoxemia, without requiring changes in respiratory support, hemodynamic status, or treatment setting. The mean time from sample collection to antibiotic therapy modification based on MSP results was 3.4 ± 2.4 h.

### 3.4. Propensity Score Matching

For the propensity score matching analysis, patients were matched based on age, sex, Horowitz index, active cancer, immunosuppression, chronic heart failure, and recent hospitalization (≥2 days within the previous 90 days). Patients with missing data for any matching variable were excluded (n = 14; none in the MSP cohort; four patients died).

Before matching, significant imbalances were observed across several covariates. After matching, all variables achieved adequate balance (standardized mean difference < 0.1), confirming the effectiveness of the matching procedure (Figure S2—Supplementary Material).

After propensity score matching, all 128 patients in the MSP cohort were retained and matched with 128 patients from the no-MSP cohort (Figure 1 and Figure S2). The mean age of the study population was 72.5 ± 15.2 years, and 111/256 patients (43.4%) were female, with no significant differences between groups. Comorbidities were evenly distributed, with no statistically significant differences (Table 1). Similarly, risk factors for multidrug-resistant organisms were comparable between the two groups (Table 2). Respiratory rate and FiO_2_ were significantly higher in the MSP group (24.5 ± 7.6 vs. 22.9 ± 6.4, *p* = 0.03; 36.2 ± 20.1 vs. 28.6 ± 16.9, *p* = 0.02), while the Horowitz index was similar between groups (Table 3). No significant differences were observed in laboratory parameters (Table S2—Supplementary Materials).

### 3.5. Primary Outcomes

The overall 30-day mortality rate was 28.9% (74/256): 50/256 (19.5%) died during hospitalization and 24/256 (9.4%) after discharge within 30 days of ED admission. The thirty-day mortality was 22.7% (29/128) in the MSP cohort, compared to the matched no-MSP cohort (35.1%, 45/128; *p* = 0.02). Kaplan–Meier analysis demonstrated significantly higher 30-day survival in the MSP cohort compared with the no-MSP cohort (log-rank Mantel–Cox *p* = 0.008) (Figure 2—right panel).

Multivariable logistic regression analysis identified independent predictors of 30-day mortality, including MSP use, comorbidities, and admission vital parameters. Early use of MSP was independently associated with reduced mortality risk (OR 0.45, 95% CI 0.20–0.99; *p* = 0.04). Among covariates, increasing age (OR 1.07 per year, 95% CI 1.03–1.11; *p* < 0.001), higher respiratory rate (OR 1.08, 95% CI 1.02–1.15; *p* = 0.013), and lower body temperature (OR 0.64, 95% CI 0.44–0.93; *p* = 0.018) were independently associated with mortality. No significant associations were observed for the remaining variables included in the model.

A Cox proportional hazards model, adjusted for demographic and clinical variables, confirmed that early use of MSP was independently associated with a reduced risk of death (HR 0.50, 95% CI 0.31–0.82; *p* = 0.006) (Table S3—Supplementary Materials).

### 3.6. Secondary Outcomes

Among secondary outcomes, a significant difference between the MSP and the no-MSP cohort was observed for thirty-day readmission rates (7.8% vs. 15.6%; *p* = 0.04); no difference was observed in LOS between the MSP and no-MSP cohorts (12.9 ± 10.0 vs. 11.0 ± 9.4 days; *p* = 0.17). Following multivariable logistic regression, also in the matched population, early MSP use was independently associated with a reduced risk of 30-day readmission (OR 0.31, 95% CI 0.11–0.90; *p* = 0.03), while no other variables (among comorbidities and vital signs) were significantly associated with that outcome.

Identification of the putative CAP-MDR etiology within the first 7 days from inclusion, considering all diagnostic methods, was significantly higher in the MSP compared with the no-MSP cohort (94.8% vs. 43.5%; *p* < 0.001).

The need for escalation of ventilatory support was significantly lower in the MSP group compared with the no-MSP group (3.9% vs. 10.6%; *p* = 0.046). No significant differences were observed in the need for initiation or escalation of vasoactive (vasopressor) support between the MSP and no-MSP groups (3.9% vs. 2.9%; *p* = 0.73).

## 4. Discussion

In this observational pre–post study, early use of MSP in the ED was associated with significantly lower 30-day mortality and readmission rates in patients with CAP-MDR, while length of stay was similar between groups. These findings suggest that rapid molecular diagnostics may improve clinically relevant outcomes when applied early in carefully selected high-risk patients.

MSPs enable rapid identification of pathogens and resistance mechanisms. Previous studies conducted in the ED, including those by Markussen et al. [10], Carturiales et al. [9], and Yu et al. [13], as well as studies performed in ICU settings [22,23,24,25], consistently demonstrated improvements in antimicrobial management but failed to show a clear benefit in clinical outcomes (Table S6—Supplementary Materials). Several factors may explain these differences.

MSP testing in our study was performed within 24 h of ED presentation, allowing optimization of antimicrobial therapy during the phase in which treatment is most likely to improve outcomes. In previous studies, timing was heterogeneous or not clearly reported, potentially reducing the observable clinical benefit because the therapeutic window may already have been missed [9,11,12,25,40]. Consistently, Kaplan–Meier curves showed that most of the survival advantage occurred within the first 3–5 days.

Following, the clinical impact of rapid molecular diagnostics is likely influenced by the type of pneumonia and the underlying epidemiology of antimicrobial resistance. Most available studies included heterogeneous populations (CAP, HAP, and VAP), in which empiric therapy is often adequate, particularly for CAP, thereby limiting the potential benefit of rapid pathogen identification [9,10,41,42]. In contrast, our study specifically focused on CAP-MDR, a population in which inappropriate empiric therapy is substantially more frequent [29]. Notably, pathogens such as Acinetobacter baumannii and carbapenemase-producing Klebsiella pneumoniae were commonly identified, reflecting the high burden of MDR organisms reported in Italy [7,29]. In this setting, earlier microbiological diagnosis is more likely to translate into meaningful clinical benefit.

Pneumonia represented the primary reason for ED presentation and the main determinant of prognosis in our cohort. Conversely, HAP and VAP often occur as complications in critically ill hospitalized patients, whose outcomes are strongly influenced by underlying illness severity. This may contribute to the lack of mortality benefit observed in ICU-based studies, where confounding by severity can obscure the effect of diagnostic interventions despite multivariable adjustment or propensity score techniques [43,44,45]. Earlier application in the ED may reduce this bias and better capture the true clinical impact of MSP.

Overall, our findings suggest that the benefit of MSP is highly context-dependent and may be maximized when two conditions are met: early testing and application to CAP patients at high risk for MDR pathogens, in whom empiric therapy is frequently inadequate. This may explain the discrepancy with previous studies and supports a more selective use of MSP in clinical practice.

The high rate of polymicrobial detection (62.5%) should be interpreted cautiously, as molecular detection does not necessarily indicate that all identified organisms are causative pathogens. In clinical practice, semiquantitative bacterial loads detected by MSP were integrated with the clinical and radiological presentation and conventional microbiological findings. Considering that semiquantitative values reported by MSP are on average 1 log_10_ higher than those reported from conventional culture, organisms detected at high abundance (10^6^ or ≥10^7^ copies/mL) were considered more likely to be clinically relevant, whereas detections at lower abundance (10^4^–10^5^ copies/mL) were interpreted more cautiously. When a low-abundance organism was the only plausible bacterial pathogen detected, antimicrobial coverage could be maintained while awaiting culture results; conversely, low-abundance detections considered more consistent with colonization did not necessarily prompt targeted therapy.

An additional limitation of MSP in polymicrobial samples is that detected resistance genes cannot always be reliably assigned to a specific organism. Their clinical interpretation should therefore consider the semiquantitative bacterial load of the concomitantly detected organisms, together with the expected genotype–phenotype relationship and subsequent phenotypic susceptibility testing.

From a clinical perspective, early identification of CAP-MDR patients in the ED may allow timely optimization of antimicrobial therapy, improving survival while supporting antimicrobial stewardship through a more targeted use of broad-spectrum antibiotics.

Nevertheless, these findings should be interpreted with caution. Given the observational pre–post design, the retrospective control group, and the non-randomized nature of the study, residual confounding, temporal bias, and measurement bias cannot be excluded. As a single-center study, the findings may have limited generalizability, and the 30-day follow-up may not capture all clinically relevant long-term outcomes. Although propensity score matching was performed, unmeasured factors such as frailty, premorbid status, or other determinants of treatment intensity may still have influenced the observed associations. However, patients with limited life expectancy were excluded by study design, and the higher rate of ventilatory support escalation observed in the no-MSP group suggests that these patients remained candidates for active treatment and full escalation of care rather than being managed with therapeutic limitations.

## 5. Conclusions

This observational pre–post study, including one prospective and one retrospective control cohort, found that the use of molecular diagnostics on respiratory samples within 24 h of hospital presentation was associated with lower 30-day mortality and 30-day readmission rates in patients with CAP-MDR. These findings suggest that early molecular diagnostics may have a clinically relevant role in the management of this high-risk population.

Confirmation in larger, adequately powered studies, ideally with randomized designs, is warranted.

## Figures and Tables

**Table 1:** Comorbidities in the General Population and Treatment Groups.

Comorbidity	General Population (n = 718)	MSP (N = 128)	No-MSP (n = 590)	*p* < 0.05	Matched Population (n = 256)	MSP (n = 128)	No-MSP (n = 128)	*p* < 0.05
Age	75.7 ± 13.8	72.1 ± 15.3	76.5 ± 13.3	**<0.01**	72.5 ± 15.2	72.0 ± 15.3	73.0 ± 15.2	0.62
Female sex	273/718 (38.0%)	51/128 (39.8%)	222/590 (37.6%)	0.69	111/256 (43.4%)	51/128 (39.8%)	60/128 (46.9%)	0.31
COPD	225/718 (31.3%)	46/128 (35.9%)	179/590 (30.3%)	0.25	88/256 (34.4%)	46/128 (35.9%)	42/128 (32.8%)	0.69
Asthma	13/718 (1.8%)	4/128 (3.1%)	9/590 (1.5%)	0.26	8/256 (3.1%)	4/128 (3.1%)	4/128 (3.1%)	1.00
HTN	463/718 (64.5%)	73/128 (57.0%)	390/590 (66.1%)	0.05	149/256 (58.2%)	73/128 (57.0%)	76/128 (59.3%)	0.80
T2DM	156/718 (21.7%)	27/128 (21.0%)	129/590 (21.8%)	0.91	52/256 (20.3%)	27/128 (21.0%)	25/128 (19.5%)	0.88
Bronchiectasis	26/718 (3.6%)	7/128 (5.4%)	19/590 (3.2%)	0.20	12/256 (4.7%)	7/128 (5.4%)	5/128 (3.9%)	0.77
CAD	151/718 (21.0%)	24/128 (18.7%)	127/590 (21.5%)	0.55	50/256 (19.5%)	24/128 (18.7%)	26/128 (20.3%)	0.87
CHF	184/718 (25.6%)	21/128 (16.4%)	163/589 (27.7%)	**<0.01**	52/256 (20.3%)	21/128 (16.4%)	31/128 (24.2%)	0.16
AF/Flutter	187/718 (26%)	24/128 (18.7%)	163/590 (27.6%)	**0.04**	52/256 (20.3%)	24/128 (18.7%)	28/128 (21.9%)	0.64
PE/DVT	64/718 (8.9%)	14/128 (10.9%)	50/590 (8.5%)	0.39	22/256 (8.6%)	14/128 (10.9%)	8/128 (6.2%)	0.26
Stroke	83/718 (11.6%)	16/128 (12.5%)	67/590 (11.3%)	0.76	29/256 (11.3%)	16/128 (12.5%)	13/128 (10.1%)	0.69
TIA	27/718 (3.8%)	2/128 (1.5%)	25/590 (4.2%)	0.20	7/256 (2.7%)	2/128 (1.5%)	5/128 (3.9%)	0.45
CKD	133/718 (18.5%)	28/128 (21.9%)	105/590 (17.8%)	0.31	49/256 (19.1%)	28/128 (21.9%)	21/128 (16.4%)	0.34
Cirrhosis	28/718 (3.5%)	6/128 (4.7%)	19/589 (3.2%)	0.42	9/256 (3.5%)	6/128 (4.7%)	3/128 (2.3%)	0.50
Immunosuppression	156/718 (21.7%)	33/128 (25.8%)	123/590 (20.8%)	0.24	64/256 (25.0%)	33/128 (25.8%)	31/128 (24.2%)	0.88
CD	283/718 (39.4%)	45/128 (35.1%)	238/590 (40.3%)	0.32	85/256 (33.2%)	45/128 (35.1%)	40/128 (31.2%)	0.60
Cancer in treatment	195/718 (27.2%)	38/128 (29.7%)	157/590 (26.6%)	0.51	74/256 (28.9%)	38/128 (29.7%)	36/128 (28.1%)	0.89

COPD: Chronic Obstructive Pulmonary Disease; HTN: Hypertension; T2DM: Type 2 Diabetes Mellitus; CAD: Coronary Artery Disease; CHF: Congestive Heart Failure; AF: Atrial Fibrillation; PE: Pulmonary embolism; DVT: Deep vein thrombosis; GERD: Gastroesophageal Reflux Disease; TIA: Transient Ischemic Attack; CKD: Chronic kidney disease; CD: Cognitive Decline.

**Table 2:** Main Risk Factors for MDR Pathogens.

Risk Factors for CAP-MDR	General Population (n = 718)	MSP (N = 128)	No-MSP (n = 590)	*p* < 0.05	Matched Population (n = 256)	MSP (n = 128)	No-MSP (n = 128)	*p* < 0.05
Hospitalized for 2 or more days within the past 90 days	346/718 (48.6%)	75/128 (58.6%)	271/590 (45.9%)	**0.01**	152/256 (59.4%)	75/128 (58.6%)	77/128 (60.1%)	0.90
Nursing home resident	186/718 (25.9%)	43/128 (33.6)	143/590 (24.2%)	**0.03**	74/256 (28.9%)	43/128 (33.6)	31/128 (24.2%)	0.13
Intravenous therapy within 30 days	320/718 (44.6%)	66/128 (51.5%)	254/590 (43.0%)	0.09	129/256 (50.4%)	66/128 (51.5%)	63/128 (49.2%)	0.80
Chemotherapy within 30 days	96/718 (13.4%)	22/128 (17.2%)	74/590 (12.5%)	0.20	35/256 (13.7%)	22/128 (17.2%)	13/128 (10.1%)	0.14
Wound care	73/718 (10.2%)	15/128 (11.7%)	58/590 (9.8%)	0.52	26/256 (10.1%)	15/128 (11.7%)	11/128 (8.6%)	0.53
Frequent hospital visitor §	439/718 (61.1%)	74/128 (57.8%)	365/590 (61.9%)	0.42	155/256 (60.5%)	74/128 (57.8%)	81/128 (63.3%)	0.44
Antibiotic therapy within 30 days	284/718 (39.6%)	62/128 (48.4%)	222/590 (37.6%)	**0.03**	126/256 (49.2%)	62/128 (48.4%)	64/128 (50.0%)	0.90
Dialysis	26/718 (3.6%)	2/128 (1.5%)	24/590 (4.0%)	0.29	6/256 (2.3%)	2/128 (1.5%)	4/128 (3.1%)	0.68
Aspiration pneumonia	116/718 (16.2%)	24/128 (18.7%)	92/590 (15.6%)	0.43	41/256 (16.0%)	24/128 (18.7%)	17/128 (13.3%)	0.30

§: Hospital visitor for medical reasons, outpatient visits, and day services.

**Table 3:** Vital Parameters at admission to the ED.

Vitals	General Population (n = 718)	MSP (N = 128)	No-MSP (n = 590)	*p* < 0.05	Matched Population (n = 256)	MSP (n = 128)	No-MSP (n = 128)	*p* < 0.05
SAP	123.6 ± 24.7	120.4 ± 26.0	124.2 ± 24.4	0.44	124.1 ± 25.9	120.4 ± 26.0	127.8 ± 25.3	0.89
DAP	70.3 ± 13.8	69.4 ± 13.5	70.6 ± 13.9	0.95	70.7 ± 13.0	69.4 ± 13.5	71.9 ± 13.1	0.82
HR	91.5 ± 22.2	94.4 ± 21.2	90.9 ± 22.3	0.74	94.4 ± 22.0	94.4 ± 21.2	94.3 ± 22.8	0.59
RR	22.5 ± 6.4	24.5 ± 7.6	22.0 ± 6.1	**<0.01**	23.7 ± 6.9	24.5 ± 7.6	22.9 ± 6.4	**0.03**
SpO_2_	92.6 ± 6.2	93.0 ± 6.0	92.5 ± 6.3	0.86	91.9 ± 6.8	93.0 ± 6.0	90.9 ± 7.5	0.09
FiO_2_	30.1 ± 18.9	36.2 ± 20.2	28.8 ± 18.3	**<0.01**	32.4 ± 19.0	36.2 ± 20.2	28.6 ± 16.9	**0.02**
HI	253.9 ± 89.9	231.5 ± 83.5	258.4 ± 90.6	**<0.01**	233.8 ± 85.8	231.5 ± 83.5	235.9 ± 88.3	0.78
BT	36.9 ± 1.4	37.2 ± 1.0	36.8 ± 1.5	0.63	37.0 ± 1.0	37.2 ± 1.0	36.8 ± 1.0	0.53
GCS	14.1 ± 2.3	14.2 ± 2.1	14.1 ± 2.3	0.55	14.2 ± 2.3	14.2 ± 2.1	14.1 ± 2.4	0.75

SAP: Systolic Arterial Pressure; DAP: Diastolic Arterial Pressure; HR: Heart Rate; RR: Respiratory Rate; FiO_2_: Fraction of Inspired Oxygen; HI: Horowitz Index; BT: Body Temperature; GCS: Glasgow Coma Scale.

## Data Availability

The data that support the findings of this study are not publicly available due to containing information that could compromise the privacy of research participants; however, the data can be made available from L.P. upon reasonable request.

## Supplementary Materials

The following supporting information can be downloaded at: https://www.mdpi.com/article/10.3390/diagnostics16193243/s1; Table S1: BIOFIRE® FILMARRAY® Pneumonia Plus Panel’s targets; Table S2: Blood Gas and Laboratory Parameters; Table S3: Only variables retained in the final multivariable model after backward stepwise selection are presented; Table S4: Conventional microbiological investigations; Table S5: Pathogens and resistance genes detected with MSP; Table S6: Literature review: Rates of modification of antibiotic therapy; Figure S1: Empiric antibiotic therapy according to local or international guidelines; Figure S2: Covariate balance before and after propensity score matching. (Ref. [46] in Supplementary Material).

## Abbreviations

The following abbreviations are used in this manuscript:

**Abbreviation**	**Definition**
BAL	Bronchoalveolar Lavage
BAS	Bronchoaspirate
CAP	Community-Acquired Pneumonia
CAP-MDR	Community-Acquired Pneumonia at Risk for Multidrug-Resistant Pathogens
CI	Confidence Interval
CRF	Case Report Form
ED	Emergency Department
ETA	Endotracheal Aspirate
FiO_2_	Fraction of Inspired Oxygen
HAP	Hospital-Acquired Pneumonia
HDU	High Dependency Unit
HFNC	High-Flow Nasal Cannula
HR	Hazard Ratio
ICU	Intensive Care Unit
MDR	Multidrug-Resistant
MSP	Molecular Syndromic Panel
NIV	Non-Invasive Ventilation
OR	Odds Ratio
PCR	Polymerase Chain Reaction
PSI	Pneumonia Severity Index
PSM	Propensity Score Matching
SD	Standard Deviation
SOFA	Sequential Organ Failure Assessment
VAP	Ventilator-Associated Pneumonia
