# Supplementary material for: Early Use of Molecular Diagnostics in the Emergency Department Reduces All-Cause Mortality in Patients with Pneumonia at Risk of Multidrug-Resistant Pathogens
Source: Diagnostics (Basel). 2026 Oct 8;16(19):3243. doi: 10.3390/diagnostics16193243 (PMC13663921; doi:10.3390/diagnostics16193243)
Supplement: Supplementary file 1 [file diagnostics-16-03243-s001.zip › diagnostics-4596417-supplementary.pdf]

MATERIAL AND METHODS

These risk factors were selected based on their established association with resistant pathogens in community-onset pneumonia [refs] and were deliberately used as an inclusive screening strategy to minimize the risk of missing patients potentially exposed to MDR pathogens. This approach was considered particularly appropriate given the high local prevalence of antimicrobial resistance and the intended early use of molecular diagnostics. Moreover, the relevance of these risk factors in our setting is supported by previous studies conducted in our local population, including HCAPPED-I [7] and the recently derived and validated RESPIRE score [Pelagatti L, De Marco M, Bosco E, Catalucci T, Gilardoni M, Mangani F, Spinicci M, Bartoloni A, Rossolini GM, Nazerian P, Vanni S. RESPIRE Score: Derivation and Validation of a New Risk Score for Prediction of Community-acquired Pneumonia Caused by Resistant Pathogens. Open Forum Infect Dis. 2026 May 21;13(6):ofag319. doi: 10.1093/ofid/ofag319. PMID: 42238377; PMCID: PMC13229114.].

STUDY POPULATION AND SETTING

The Departments of Emergency Medicine and HDU were responsible for patients' assessment and enrolment, respiratory sample collection, and data acquisition in the prospective cohort. They also identified all pneumonia cases, selected eligible patients with CAP-MDR, and managed enrolment, data collection, and informed consent procedures for the retrospective cohort. The Microbiology and Virology Unit processed the lower respiratory samples, including BAL, BAS and ETA for microbiological analysis by MSP testing (in the prospective cohort) and by standard procedures including conventional cultures, urinary antigen testing for Legionella and pneumococci, and additional analyses (blood cultures, molecular and conventional assays for fungal pathogens, respiratory viral testing by molecular assays or rapid antigen-based methods) if deemed appropriate. The Infectious and Tropical Diseases Unit assessed the appropriateness of antimicrobial therapy based on MSP results and evaluated cases of antibiotic escalation and de-escalation.

Patients in the prospective cohort were enrolled at the time of respiratory sample collection, provided they met inclusion criteria. Patients in the retrospective cohort were initially identified through a dedicated statistical extraction system (BI4), including all individuals discharged, deceased, or admitted following ED evaluation during the study period with a coded or textual diagnosis of pneumonia. Among all patients with pneumonia, only those with CAP-MDR were retained for inclusion if they met inclusion criteria.

All patients were followed for 30 days from hospital admission. Collected data included demographic characteristics; medical history, including comorbidities and risk factors for multidrug-resistant infection; vital signs at emergency department presentation; laboratory and arterial blood gas results; microbiological findings; antibiotic therapy; hemodynamic support; invasive and non-invasive mechanical ventilation; clinical outcomes; level of care; and in-hospital complications. Data were extracted from electronic medical records (First Aid, Archimed, DnWeb) or obtained through telephone follow-up when necessary.

Objective - Safety outcomes

Complications related to respiratory samples acquisition

Major complications related to bronchoalveolar lavage (BAL) and bronchial aspiration are defined as events requiring escalation of care or resulting in clinically significant deterioration. These include severe hypoxemia requiring increased ventilatory support or intubation, hemodynamic instability necessitating vasoactive support, clinically relevant bleeding, cardiac arrhythmias requiring treatment, pneumothorax, or procedure-related death.

Minor complications are defined as transient and self-limiting events not requiring changes in clinical management. These include cough, transient hypoxemia not requiring escalation of respiratory support, mild bleeding, cough, bronchospasm responsive to treatment, and transient hemodynamic fluctuations without the need for therapeutic intervention.

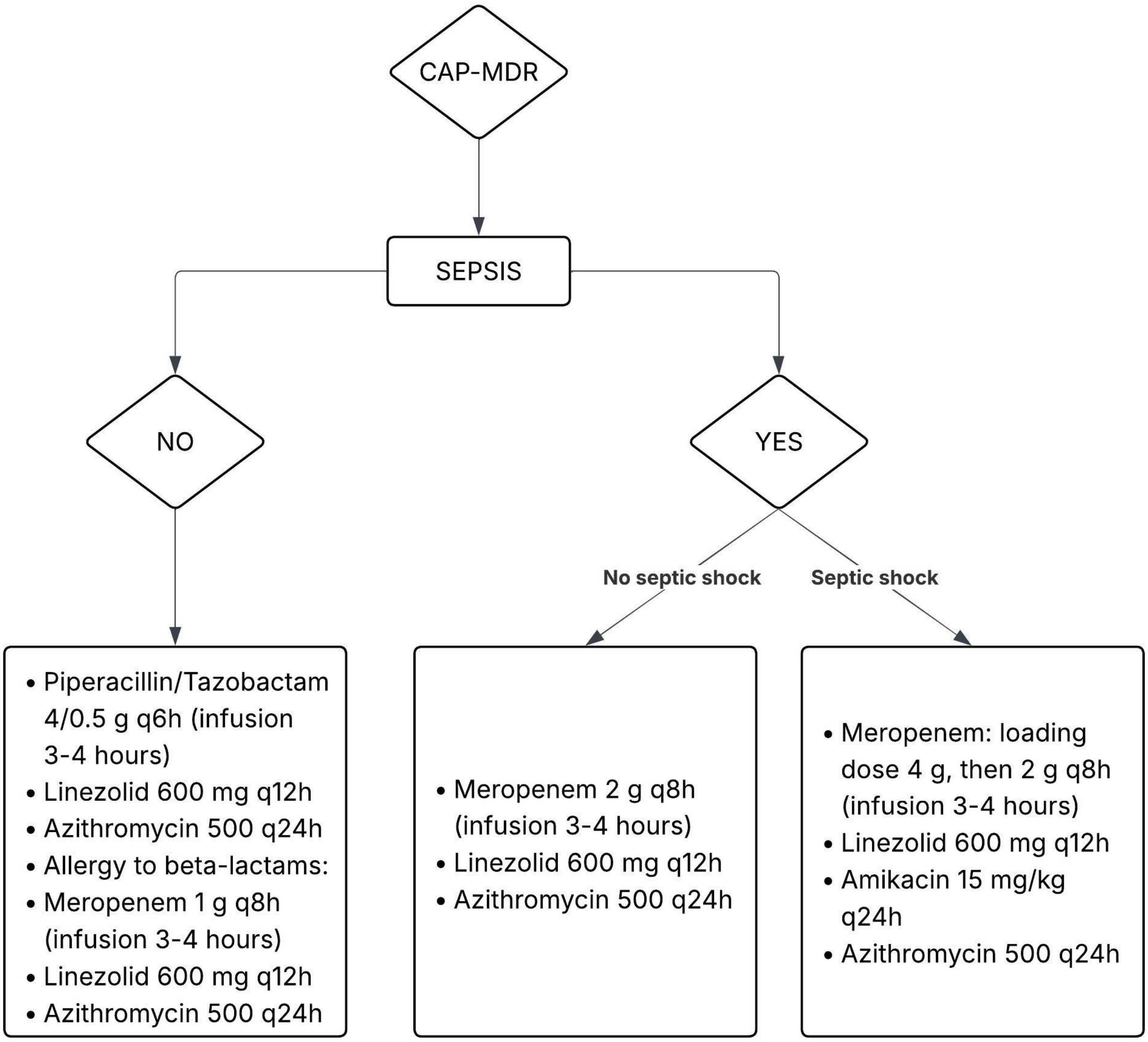

Figure S1. Empiric antibiotic therapy according to local or international guidelines.

MICROBIOLOGICAL INVESTIGATIONS

INDEX TEST: PCR MULTIPLEX (BIOFIRE® FILMARRAY® Pneumonia Plus Panel)

Respiratory samples (BAL, sputum, ETA) were analyzed using syndromic molecular panel specific for community-acquired pneumonia (CAP) and healthcare-associated pneumonia (HCAP), the BIOFIRE® FILMARRAY® Pneumonia Plus Panel. The BIOFIRE® FILMARRAY® Pneumonia Plus Panel is a multiplex nested real-time PCR certified by the FDA, CE-IVD, and TGA that in a self contained pouch can identify 33 targets of which 18 bacterial pathogens (including 11 Gram-negative, 4 Gram-positive, and 3 atypical organisms), 7 antibiotic resistance genes, and 9 respiratory viruses (Table S1) with a turnaround time (TAT) of about 1 hour.

Results for typical bacterial pathogens is reported semiquantitatively , as genomic copies/ml.

The overall sensitivity and specificity for BAL samples are 96.2% and 98.3%, respectively; for sputum samples, sensitivity and specificity are 96.3% and 97.2%, respectively.

The system integrates sample preparation, nucleic acid extraction and purification, amplification, detection, and analysis within a single platform and requires approximately 2 minutes of hands-on time.

Results were communicated by telephone to the treating physicians. The Microbiology Department did not provide therapeutic recommendations. Conventional culture testing (gold standard) was performed in parallel on all respiratory samples.

**Bacteria (Semi-Quantitative)**	**Atypical Bacteria (Qualitative)**	**Viruses**	**Antimicrobial Resistance Genes**
Acinetobacter calcoaceticus-baumannii complex<br>*Enterobacter cloacae*<br>*Escherichia coli*<br>*Haemophilus influenzae*<br>*Klebsiella aerogenes*<br>*Klebsiella oxytoca*<br>*Klebsiella pneumoniae group*<br>*Moraxella catarrhalis*<br>*Proteus spp.*<br>*Pseudomonas aeruginosa*<br>*Serratia marcescens*<br>*Staphylococcus aureus*<br>*Streptococcus agalactiae*	*Legionella pneumophila*<br>*Mycoplasma pneumoniae*<br>*Chlamydia pneumoniae*	*Influenza A*<br>*Influenza B*<br>*Adenovirus*<br>*Coronavirus (non SARS-coV)*<br>*Parainfluenza virus*<br>*Respiratory Syncytial Virus*<br>*Human Rhinovirus/Enterovirus*<br>*Human Metapneumovirus*<br>*MERS-CoV*	*CTX-M*<br>*KPC*<br>*NDM*<br>*Oxa48-like*<br>*VIM*<br>*IMP*<br>*mecA/C and MREJ*

Streptococcus pneumoniae<br>*Streptococcus pyogenes*			

Table S1. BIOFIRE® FILMARRAY® Pneumonia Plus Panel's targets.

Other methods for pathogens identification:

Blood cultures:

Blood cultures were collected in specific blood bottles cultures media (BD BACTEC™ Lytic Anaerobic and BD BACTEC™ Plus Aerobic – BD Sparks, USA) and incubated in blood cultures incubation system BACTEC™FX (BD Sparks, USA) until they flag positive. From blood bottles flagged positive, an aliquot of 1-2ml is manually transferred into Copan's BC+™ vacuum tube (Copan Italia, IT) and loaded into the WASPLab (Copan Italia, IT) instrument that automatically handles agar plates streaking and Gram slide preparation. For agar plates streaking, 90 microliters of blood is inoculated onto non-selective media such as Chocolate agar, and 10 microliters on chromogenic agar (ChromID CPSElite-Biomerieux, FR) and incubated at 37°C in a 5% CO_2 atmosphere for Chocolate agar and 37°C atmosphere for chromogenic agar . Bacterial growth is preliminary assessed after 6 hours of incubation and again after 24 hours of incubation. Bacterial colonies identification is performed with MALDI-ToF (MALDI Biotyper®, Bruker, Germany) and antimicrobial susceptibility testing (AST) is performed by automated systems such as BD Phoenix™ (BD Sparks, USA), Vitek®2 (bioMérieux) or

commercial broth microdilution (Merlin Diagnostika GmbH, Germany) according with manufacturer instructions.

Urinary Antigens:

Urinary antigen tests for *Streptococcus pneumoniae* and *Legionella pneumophila* were performed by fluorescence immunoassay in urine samples (STANDARD F *S.pneumoniae* and *Legionella* Ag FIA, SD BIOSENSOR)

Conventional respiratory cultures:

Lower respiratory tract (LRT) samples were processed using conventional culture methods for the detection of bacterial and fungal pathogens. Ten microliters of respiratory samples fluid were inoculated onto non-selective media, including blood agar, CHROMagar™ Candida incubated at 37° and chocolate agar , incubated at 37 °C in a 5% CO_2 atmosphere. Microbial growth was assessed after 36 and 48 hours of incubation.

Bacterial colonies were identified by matrix-assisted laser desorption/ionization time-of-flight mass spectrometry (MALDI-TOF MS- MALDI Biotyper®, Bruker, US). Potential pathogens were reported semi-quantitatively as colony-forming units per milliliter (CFU/mL). Antimicrobial susceptibility testing (AST) was performed using automated systems such as BD Phoenix (Bruker US), Vitek®2 (bioMérieux) or commercial broth microdilution (Merlin Diagnostika GmbH, Germany).

Molecular methods for fungal pathogens identification:

For fungal pathogens identification quantitative PCR assays were performed on respiratory specimens (BAL, tracheal aspirate, or sputum) to detect DNA from *Aspergillus spp.* and *Pneumocystis jirovecii* by using automated platform singleplex RT-PCR (Elite MGB® Kit Elite Ingenius- ElithechGroup).

Galattomannan test:

Galactomannan test in serum and respiratory samples was performed by chemiluminescent immunoassay (Monotest Virclia- Vircell SL).

Molecular methods for viral pathogens identification:

For the detection of viral pathogens (e.g. *SARS-CoV-2, Herpesviruses*) nucleic acid amplification tests were performed by automated platform singleplex RT-PCR (Elite MGB® Kit Elite Ingenius- ElithechGroup and Allplex™SARS-CoV-2- Seegene) according to manufacturer's instructions.

Regarding *SARS-CoV-2* nasopharyngeal swabs were processed by chemiluminescent enzyme immunoassay tests (CLEIA- Lumipulse® SARS-CoV-Ag- Fujirebio and immunochromatografic point of care tests directly in emergency department (Alifax srl, Fiatest).

RESULTS

ABG and laboratory	General population (n=718)	MSP (N=128)	No-MSP (n=590)	p<0,05	Matched population (n=256)	MSP (n=128)	No-MSP (n=128)	p<0,05
pH	7.41±0.29	7.36±0.68	7.42±0.09	**<0.01**	7.39±0.48	7.36±0.68	7.41±0.1	0.18
pO2 (mmHg)	68.1±33.4	73.7±50.9	67.0±28.5	**<0.01**	73.7±42.0	73.7±50.9	73.6±31.0	0.56
pCO2 (mmHg)	39.39±13.8	37.2±18.2	39.9±12.6	**<0.01**	39.1±16.2	37.2±18.2	39.1±13.7	0.06
HCO3 (Mmol/L)	25.3±5.7	23.9±8.5	25.7±4.8	**<0.01**	24.3±7.2	23.9±8.5	24.7±6.5	0.07
Lac (mEq/L)	1.8±1.8	1.7±1.6	1.8±1.9	0.34	1.8±1.7	1.7±1.6	1.9±1.8	0.26
Hb (g/dL)	11.6±2.2	11.7±2.2	11.6±2.3	0.98	11.7±2.4	11.7±2.2	11.6±2.5	0.34
WBC (x 10^9/L)	11.9±7.1	12.3±8.5	11.8±6.8	0.06	12.0±7.8	12.3±8.5	11.7±7.1	0.39
Platelet (x 10^9/L)	259.1±141.8	269.7±129.3	256.8±144.4	0.99	268.6±132.1	269.7±129.3	267.6±135.4	0.89
CRP (mg/L)	108.3±96.0	112.8±98.1	107.3±96.0	0.69	110.6±100.8	112.8±98.1	108.4±106.8	0.62
Procalcitonin (ng/mL)	6.1±30.6	7.0±37.2	5.9±28.7	0.55	5.7±30.0	7.0±37.2	4.2±18.6	0.20
INR	1.3±0.6	1.3±0.4	1.4±0.7	0.06	1.3±0.6	1.3±0.4	1.4±0.9	0.20
Creatinine (mg/dL)	1.4±1.2	1.2±0.9	1.5±1.2	0.11	1.3±1.0	1.2±0.9	1.3±1.0	0.80
Sodium (mEq/L)	139.8±7.8	138.1±6.3	140.2±8.1	0.18	139.2±7.0	138.1±6.3	140.4±7.5	0.30

Potassium (mEq/L)	4.3±0.8	4.2±0.7	4.3±0.8	0.13	4.3±0.7	4.2±0.7	4.4±0.8	0.33
Glucose (mg/dL)	142.8±70.7	142.5±58.2	142.8±73.4	0.49	139.9±56.9	142.5±58.2	136.8±55.6	0.86
ALT (U/L)	34.1±68.1	29.7±47.8	35.1±71.9	0.42	34.2±53.4	29.7±47.8	38.9±50.6	0.20

Table S2. **Blood Gas and Laboratory Parameters.** WBC: White Blood Cell count; CRP: C-reactive Protein; PT; INR: International Normalized Ratio; ALT: Alanine Transaminase.

Variable	General population (n=718)<br>HR (95% CI)	p<0,05	Matched population (n=256)<br>HR (95% CI)	p<0,05
MSP	0.60 (0.36–0.99)	0.04	0.50 (0.31–0.82)	0.006
Age	1.05 (1.03 – 1.06)	<0.001	1.04 (1.01- 1.06)	0.002
RR	1.04 (1.01-1.06)	0.002	1.04 (1.01-1.08)	0.04
SBP	1.01 (1.01-1.02)	<0.001	-	-
Active cancer	-	-	1.52 (1.32-1.85)	0.01

Table S3. Only variables retained in the final multivariable model after backward stepwise selection are presented. Variables included in the initial multivariable model were the following: MSP; age; sex; Horowitz index; active cancer; CHF; HR; SBP; RR; previous hospitalization; nursing home resident. The variable MSP was retained in the final multivariable model. CI, confidence interval; HR, hazard ratio.

Variable	Overall MSP (n=128)	Overall No-MSP (n=590)	p value	Matched MSP (n=128)	Matched No-MSP (n=128)	p value
**Conventional microbiological investigations**						
Blood cultures performed	71/128 (55.5%)	124/590 (21.0%)	<0.001	71/128 (55.5%)	27/128 (21.1%)	<0.001
Blood cultures positive	12/128 (9.4%)	37/590 (6.3%)	0.207	12/128 (9.4%)	8/128 (6.2%)	0.352
Urinary antigen for Legionella performed	94/128 (73.4%)	211/590 (35.8%)	<0.001	94/128 (73.4%)	51/128 (39.8%)	<0.001
Urinary antigen for Legionella positive	2/128 (1.6%)	3/590 (0.5%)	0.162	2/128 (1.6%)	1/128 (0.7%)	0.498
Urinary antigen for S. pneumoniae performed	95/128 (74.2%)	207/590 (35.1%)	<0.001	95/128 (74.2%)	50/128 (39.1%)	<0.001
Urinary antigen for S. pneumoniae positive	8/128 (6.2%)	26/590 (4.4%)	0.080	8/128 (6.2%)	6/128 (4.7%)	0.324

Table S4. **Conventional microbiological investigations.**

**Pathogen**	**N (n=128)**	**10^4**	**10^5**	**10^6**	**10^7**
Bacteria					
Staphylococcus aureus	30	7	12	6	5
Klebsiella pneumoniae	29	12	2	3	12
Pseudomonas aeruginosa	20	3	1	4	12
Haemophilus influenzae	19	4	1	3	11
Acinetobacter calcoaceticus-baumannii complex	12	2	1	2	7
Proteus spp.	10	2	4	1	3
Escherichia coli	9	4	1	0	4
Enterobacter cloacae complex	7	3	4	0	0
Moraxella catarrhalis	5	2	1	1	1
Streptococcus pneumoniae	5	0	0	0	5
Serratia marcescens	4	0	2	1	1
Streptococcus agalactiae	4	1	0	2	1
Klebsiella oxytoca	2	1	1	0	0
Virus					
Influenza A	10	-	-	-	-
Rhinovirus/Enterovirus	10	-	-	-	-
Coronavirus	3	-	-	-	-
Metapneumovirus	2	-	-	-	-
Atypical Bacteria					
Legionella pneumophila	1	-	-	-	-
Resistance Genes					
CTX-M	14	-	-	-	-
CTX-M + KPC	12	-	-	-	-

mecA/C	10	-	-	-	-
CTX-M + mecA/C	3	-	-	-	-
CTX-M + KPC + mecA/C	2	-	-	-	-
VIM	2	-	-	-	-
KPC + mecA/C	1	-	-	-	-
CTX-M + KPC + NDM + OXA-48-like	1	-	-	-	-

Table S5. Pathogens and resistance genes detected with MSP.

Author	Year	Design	Setting	Pneumonia	Number of patients	MDR prevalence in entire cohort	Rate of change in therapy in MSP group	Mortality (%)
Pelagatti et al [7]	2025	Prospective single-center observational cohort (single-arm)	ED	CAP-MDR	93	37.6%	65.6% (escalation 44.1%, de-escalation 21.5%)	30-day mortality: 25.8%
Markussen et al [10]	2024	RCT - parallel-arm, single-blinded, single-center, superiority trial	ED	CAP	374 (187 each arm)	Not reported	Rate of change in therapy 35.3%	30-day mortality: 4.8% (MSP) vs 3.7% (no MSP), p=0.61
Markussen et al [14]	2024	Prospective observational study	ED	CAP	384	Not reported	NA	90-day mortality: 7.8%
Carturiales et al [9]	2022	Multicentre randomized controlled trial (open-label, parallel-group)	ED	CAP	294 (148 MSP vs 146 standard care)	Not reported	Targeted therapy - 57.7% vs 24.1% (4h) - 65.4% vs 34.5% (48h)	30-day mortality: 3.6% (MSP) vs 2.7% (no MSP), p=0.74
Yu et al [13]	2025	Prospective, single-center, quasi-randomized controlled study with time-based allocation	ED	CAP	282	Not reported	Reduced antibiotic changes within 72h: 14.7% vs 28.4%, p=0.046	30-day mortality: Spring 4.2% vs 6.8%, p = 0.47; Autumn–Winter: 5.3% vs 9.5%, p = 0.35
Giacobbe et al [12]	2025	Multicentre prospective observational study	ICU	VAP	237 (132 panel vs 105 culture)	Resistance genes detected in 26.5%	De-escalation OR early appropriate therapy: 41.3% vs 27.8%, p = 0.041. De-escalation: 48.0% vs 24.2%, p = 0.030	30-day mortality: 28.7% vs 26.4%, p = 0.94

Enne et al [15]	2025	Multicentre randomized controlled trial (open-label, pragmatic)	ICU	HAP, VAP	545 (MSP vs standard care)	Not reported	Improved appropriate/proportionate therapy at 24h: 76.5% vs 55.9%	28-day mortality: 31.3% vs 28.2%, p>0.05
Velly et al [16]	2022	Prospective, single-center, open-label randomized controlled trial	ED	CAP	451 (226 SOC vs 225 MSP)	Non reported	Non reported	28-day mortality: 6.0% vs 4.7%, p = 0.54
de Albuquerque Pessoa Dos Santos et al [11]	2025	Systematic review and meta-analysis of randomized controlled trials	ICU	CAP, HAP, VAP	2466 (5 RCTs)	Not reported	Adequacy of initial antimicrobial therapy: RR 1.82 (95% CI 1.10–3.00, p = 0.02	In-hospital mortality: RR 1.04, 95% CI 0.90–1.21, p = 0.57
Stafylaki et al [17]	2022	Retrospective observational study with retrospective control	ICU	CAP, HAP, VAP	119 (79 MSP vs 40 control)	20.3% XDR*	Not reported**	Overall mortality during hospitalization/ICU stay: MSP 20.25% vs Control 20%, p > 0.99
Aissauoi et al [18]	2025	Prospective multicenter observational study	ICU	CAP, HAP, VAP		41.9% (88/210)§	Any modification: 58%; De-escalation/cessation: 11% ; Escalation: 26.5%; Adequacy: 7.5%; Initiation: 13%	ICU mortality not directly compared; appropriate therapy associated with reduced mortality (aOR 0.37, p=0.038)

Ellekvist et al [19]	2025	Prospective randomized controlled trial	ED	CAP	118 (64 intervention vs 54 control)	Not reported	No difference: 28% vs 30% (NS)	30-day mortality: 9.4% vs 7.5% (p = not reported; NS)
Darie et al [20]	2022	Multicentre, open-label randomized controlled trial	Mixed wards, ICU	CAP, HAP	208 (100 intervention vs 108 control)	Not reported	Reduced duration of inappropriate antibiotic therapy: 47.1 h vs 85.7 h, p < 0.0001	8% vs 10% , p not reported (no significant difference)
Virk et al [24]	2024	Single-center, open-label, pragmatic randomized controlled trial	Hospital (mixed wards/ ICU)	CAP + HAP + VAP	1152 (563 intervention vs 589 control)	Not reported	Any antibiotic modification: 65.2% vs 68.4% (p=0.24)	30-day mortality: 22.6% vs 20.4% (p=0.42)

*Table S6. Literature Review: Rates of modification of antibiotic therapy. aOR, adjusted odds ratio; CAP, community-acquired pneumonia; CI, confidence interval; DDD, defined daily dose; ED, emergency department; HAP, hospital-acquired pneumonia; ICU, intensive care unit; MDR, multidrug-resistant; MSP, molecular syndromic panel; NA, not available; NS, not significant; OR, odds ratio; RCT, randomized controlled trial; RR, relative risk; SOC, standard of care; VAP, ventilator-associated pneumonia; XDR, extensively drug-resistant. * only XDR prevalence reported; no overall MDR prevalence provided. *** Rates of antibiotic modification were not uniformly reported; therefore, surrogate measures such as antimicrobial consumption (DDD) or qualitative assessments of de-escalation were used when quantitative data were unavailable. §Resistance genes identified.

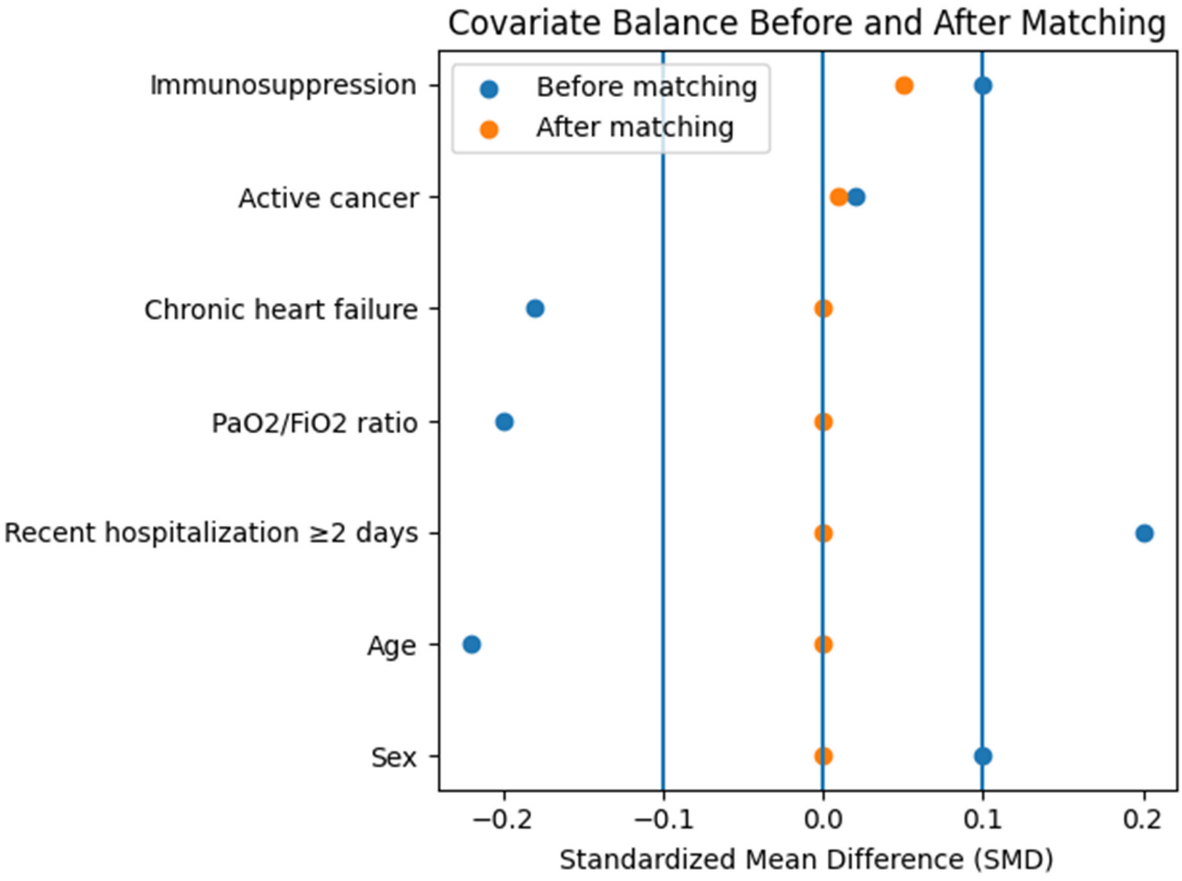

Figure S2. Covariate balance before and after propensity score matching.
